# Chronic administration of palmitoleic acid reduces insulin resistance and hepatic lipid accumulation in KK-A^y ^Mice with genetic type 2 diabetes

**DOI:** 10.1186/1476-511X-10-120

**Published:** 2011-07-21

**Authors:** Zhi-Hong Yang, Hiroko Miyahara, Akimasa Hatanaka

**Affiliations:** 1 Central Research Laboratory, Tokyo Innovation Center, Nippon Suisan Kaisha, Ltd., 32-3 Nanakuni 1 Chome Hachioji, Tokyo 192-0991, Japan

## Abstract

**Background:**

Studies have demonstrated the beneficial effect of palmitoleic acid (C16:1 n-7) on reducing muscle insulin resistance and preventing beta-cell apoptosis. However, the effect of palmitoleic acid on diabetes remains to be elucidated. The aim of this study was to examine the antidiabetic effect of palmitoleic acid in KK-A^y ^mice, a spontaneous model for studies of obese type 2 diabetes with low insulin sensitivity.

**Methods:**

KK-A^y ^mice were orally administered vehicle, 300 mg/kg of palmitoleic acid, or 300 mg/kg of palmitic acid (C16:0) on a daily basis for 4 weeks.

**Results:**

Palmitoleic acid reduced body weight increase, ameliorated the development of hyperglycemia and hypertriglyceridemia, and improved insulin sensitivity. In addition, hepatic characteristics were significantly affected, as weight of the liver and hepatic triglyceride levels were lower in the palmitoleic acid group when compared to the control (vehicle and palmitic acid groups). Oil red O staining clearly indicated reduced hepatic lipid accumulation in response to palmitoleic acid. Furthermore, palmitoleic acid down-regulated mRNA expressions of proinflammatory adipocytokine genes (*TNFα *and *resistin*) in white adipose tissue and lipogenic genes (*SREBP-1*, *FAS*, and *SCD-1*) in liver.

**Conclusions:**

These results suggest that palmitoleic acid improves hyperglycemia and hypertriglyceridemia by increasing insulin sensitivity, in part owing to suppressing proinflammatory gene expressions and improving hepatic lipid metabolism in diabetic mice.

## Background

Type 2 diabetes mellitus, a worldwide health issue, is a cluster of metabolic diseases characterized by hyperglycemia that result from defects in insulin secretion or/and action [1]. Numerous lines of evidence support the involvement of fatty acids in type 2 diabetes mellitus, and many studies have demonstrated that fatty acids with different degrees of saturation have different effects on insulin sensitivity and glucose/lipid metabolism. Saturated fatty acids are key contributors to insulin resistance, whereas unsaturated fatty acids have beneficial effects and improve diabetes pathologies through multiple mechanisms [2,3]. Palmitoleic acid (C16:1) is an omega-7 monounsaturated fatty acid that is abundant in plant and marine sources [4-6]. It has been demonstrated that palmitoleic acid prevents beta-cell apoptosis induced by glucose or saturated fatty acids [7,8], and diets rich in palmitoleic acid improve circulating lipid profile in both animal model [9] and human subjects [10,11]. Furthermore, a recent report demonstrated that palmitoleic acid functions as an adipose tissue-derived lipid hormone that stimulates muscle insulin action and suppresses hepatosteatosis in mice deficient in fatty acid binding protein [12]. Very little data are available, however, concerning the effects of palmitoleic acid on animal models of diabetes.

KK-A^y ^mice are widely used as a model for type 2 diabetes [13]. Compared with KK mice, the strain with the Ay allele (KK-A^y^) are obese and develop severe, early-onset hyperinsulinemia, hyperglycemia, hypertriglyceridemia, and fatty liver. In the present study, we examined the effect of palmitoleic acid on diabetic risk factors in KK-A^y ^mice, and further investigated the molecular mechanisms underlying these effects.

## Methods

### Materials

Palmitic acid (C16:0) and palmitoleic acid (C16:1 n-7) in the form of free fatty acids were purchased from Sigma Chemical Company (St. Louis, U.S.A.) and stored at -20°C until use. Polyglycerol ester was obtained from Mitsubishi-Kagaku Foods Corporation (Tokyo, Japan). The fatty acids were dispersed in 1.5% (w/w) of polyglycerol ester aqueous solution (vehicle) by sonification to a final concentration of 30 mg/mL. In the preliminary test, the mean particle diameter and size distribution of fatty acid solutions were measured using a laser diffraction particle size analyser (LS 13320, Beckman Coulter Ltd, Miami FL, USA). All measurements were repeated twice, and no marked differences in emulsion particle sizes between palmitic acid and palmitoleic acid solution were detected (data not shown).

### Animals

Six-week-old male KK-A^y ^mice (SLC, Shizuoka, Japan) were housed individually in stainless wire mesh cages in a room with a 12-h light/dark cycle and a constant temperature of 24 ± 1°C. Mice had free access to distilled water and laboratory chow (Labo MR Stock, Nosan Corporation, Japan) for 1 week to stabilize metabolic conditions. After the stabilization period, 30 mice were randomly divided into 3 equal groups and orally administered either 1.5% (w/w) of polyglycerol ester (vehicle), 300 mg/kg of palmitic acid solution (palmitic acid group), or 300 mg/kg of palmitoleic acid solution (palmitoleic acid group). These dietary supplements were provided through a stomach tube each morning between 10:00 and 11:00 for 4 weeks. All mice were allowed free access to water and chow during the experimental period. We measured each mouse's food intake (every 3-4 days) and body weight (once per week) throughout the experiment. This study was conducted in compliance with the National Institutes of Health: Guide for the Care and Use of Laboratory Animals, and the Institutional Animal Care and Use Committee of Japan SLC Inc. approved the protocol.

### Insulin tolerance test

To evaluate effect of palmitoleic acid on insulin sensitivity, an insulin tolerance test was performed on day 22. Following 5 h of food deprivation, an insulin solution (0.5 U/kg body weigh, Humulin R U-100, Eli Lilly, Japan) was administered subcutaneously. Blood samples were collected immediately preceding insulin administration (0 min), and 30, 60, and 120 min later. Blood samples were centrifuged at 2,000 × *g *for 15 min, and plasma glucose concentrations were measured using a glucose test kit (Glucose CII-test, Wako Pure Chemical Industries, Ltd., Japan).

### Blood analysis

To determine plasma glucose levels, blood samples were taken from the retro-orbital venous plexus of each mouse once per week. Samples were drawn from animals in a non-fasted state, immediately preceding administration of the testing materials. On the final day of the experiment, animals were deprived overnight food. The next morning, mice were anesthetized using diethyl ether, and sacrificed by bleeding from the postcaval vein. The blood was collected, and levels of plasma triglyceride, total cholesterol, and free fatty acids were measured using enzyme assay kits (Triglycerol E-Test, Cholesterol E-Test, and NEFA C-Test reagents, respectively; Wako). Plasma insulin concentrations were determined using reagents from an insulin ELISA kit (Morinaga Institute of Biological Science, Inc., Japan).

### Organ measurements

On the final day of the study, vital organs were dissected and weighed after a short wash in cold phosphate-buffered saline (PBS, pH 7.4). Mesenteric adipose tissue and livers were maintained at -80°C until analyzed.

### Liver triglyceride contents analysis

Liver total lipids were extracted from a portion of the tissue sample as described by Folch *et al*. [14]. Extracted lipids were dried using a vacuum concentrator (Concentrator Plus 5305, Eppendorf) and dissolved in 2-propanol containing 10% (w/w) Triton X-100. Triglyceride concentrations were determined using Wako Triglyceride E-Test.

### Oil red O staining of liver sections

Liver samples were washed in cold PBS and fixed overnight at 4°C with a 4% paraformaldehyde solution in PBS. Samples were embedded in optimal cutting temperature compound (Tissue-Tek, Sakura Finetek Japan Co., Ltd.) and frozen at -80°C. Six-μm thick sections were cut using a cryotome (Jung Frigocut model 2800N, Leica) at -25°C. Sections were stained for 20 min with fresh oil red O solution (Sigma). Stained liver sections were washed thoroughly with distilled water prior to microscopic observation. Images were captured using an Olympus camera mounted on an Olympus upright microscope.

### Real-time polymerase chain reaction (RT-PCR)

Mesenteric adipose tissue and liver total RNA were extracted with TRIzol reagent (Qiagen, Valencia, CA), and cDNAs were synthesized from total RNA (1 μg) by using a PrimeScript 1^st^-strand cDNA synthesis kit (TaKaRa Bio, Otsu, Japan) following manufacturer's protocols. To quantify mRNA expression levels, the PCR amplifications of different cDNAs were performed with SYBR premix Ex Taq (TaKaRa Bio) on the Applied Biosystems 7300 Real-Time PCR System (Life Technologies Co., Japan) according to the manufacturer's instructions. The expression levels of target gene were normalized by the expression of the endogenous control gene encoding 18S ribosomal RNA. The primer sequences used were shown in Table 1.

**Table 1:** GenBank accession numbers and primer sequences used in PT-PCR.

Gene	Primer Sequences	Accession Number
SREBP-1	5'-GATGTGCGAACTGGACACAG-3'	NM_011480
	5'-CATAGGGGGCGTCAAACAG-3'	
FAS	5'-GGAGGTGGTGATAGCCGGTAT-3'	NM_007988
	5'-TGGGTAATCCATAGAGCCCAG-3'	
SCD-1	5'-TTCTTGCGATACACTCTGGTGC-3'	NM_009127
	5'-CGGGATTGAATGTTCTTGTCGT-3'	
TNFα	5'- CCCTCACACTCAGATCATCTTCT-3'	NM_013693
	5'-GCTACGACGTGGGCTACAG-3'	
Resistin	5'- AAGAACCTTTCATTTCCCCTCCT-3'	NM_022984
	5'-GTCCAGCAATTTAAGCCAATGTT-3'	
Adiponectin	5'-TGTTCCTCTTAATCCTGCCCA -3'	NM_009605
	5'-CCAACCTGCACAAGTTCCCTT-3'	

*SREBP-1*: Sterol regulatory element binding protein 1, *FAS*: Fatty acid synthase, *SCD-1*: Stearoyl CoA desaturase-1, *TNFα*: Tumor necrosis factor alpha.

### Statistical Analysis

Data are expressed as mean ± standard errors (SE). Statistical analyses with Tukey multiple comparisons test were performed to determine differences between groups. The results were considered to be significant if the value of *p *was < 0.05.

## Results

### Effect of palmitoleic acid on body and organ weights

As shown in Table 2, administration of palmitoleic acid to KK-A^y ^mice caused a significant decrease (*p *< 0.05) in body and liver weights, as well as a marked increase (*p *< 0.05) in pancreas weight as compared with the palmitic acid or control group. Mesenteric white adipose tissue, brown adipose tissue, and skeletal muscle, however, did not differ significantly among the three groups. In addition, food intake of the palmitoleic acid group was lower than in the control group (*p *< 0.05), whereas palmitic acid did not affect food intake.

**Table 2:** Body weights, food intakes, organ weights and plasma lipid levels.

	Control	C16:0	C16:1
Initial body weight (g)	30.0 ± 0.3	30.1 ± 0.3	30.8 ± 0.4
Final body weight (g)	36.7 ± 0.5^a^	35.3 ± 0.7^a^	32.1 ± 1.1^b^
Food intake (g/day)	6.1 ± 0.1^a^	5.8 ± 0.2^a^	5.1 ± 0.3^b^

Organ weights			
Liver (mg/g BW)	46.9 ± 1.3^a^	46.3 ± 1.3^a ^	40.2 ± 0.9^b^
Pancreas (mg/g BW)	9.6 ± 0.2^a ^	10.2 ± 0.2^a ^	11.2 ± 0.3^b^
Mesenteric WAT (mg/g BW)	21.2 ± 0.9	21.1 ± 0.4	19.1 ± 1.2
BAT (mg/g BW)	7.2 ± 0.5	7.1 ± 0.4	7.6 ± 0.4
Skeletal muscle (mg/g BW)	8.8 ± 0.3	8.6 ± 0.3	9.1 ± 0.5

Plasma lipid parameters			
Triglyceride (mg/dL)	259.2 ± 21.1^a ^	237.3 ± 15.7^a ^	129.1 ± 23.7^b^
Total cholesterol (mg/dL)	112.7 ± 8.2	99.4 ± 5.9	109.7 ± 4.6
Free fatty acid (mEq/L)	0.49 ± 0.03	0.48 ± 0.02	0.48 ± 0.03

KK-A^y ^mice were orally administrated vehicle (control), 300 mg/kg of palmitic acid, or 300 mg/kg palmitoleic acid on a daily basis for 4 weeks. Values are means ± SE, n = 10. Means in a row with superscripts without common letter are different, *p *< 0.05. C16:0, palmitic acid; C16:1, palmitoleic acid; BW, body weight; WAT, white adipose tissue; BAT, brown adipose tissue.

### Effect of palmitoleic acid on plasma glucose level

**Figure 1:**
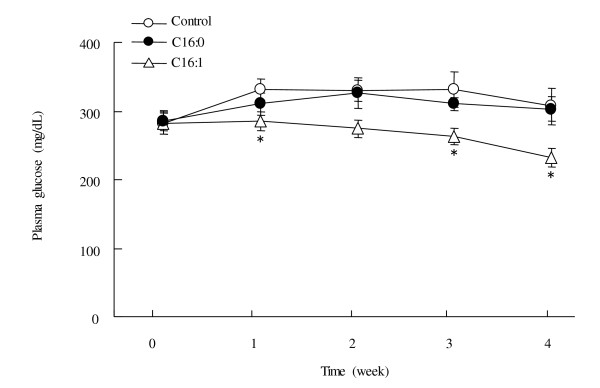
**Effect of palmitoleic acid on plasma glucose concentrations in KK-A^y ^mice**. Animals were orally administered vehicle (control), 300 mg/kg of palmitic acid, or 300 mg/kg palmitoleic acid on a daily basis for 4 weeks. Values are means ± SE, n = 10. *p < 0.05 compared with the control group. C16:0, palmitic acid; C16:1, palmitoleic acid.

Palmitoleic acid administration significantly decreased (*p *< 0.05) plasma glucose levels compared to animals administered the vehicle alone (Figure 1). In contrast, plasma glucose concentrations did not differ between the palmitic acid and control groups.

### Effect of palmitoleic acid on insulin sensitivity

**Figure 2:**
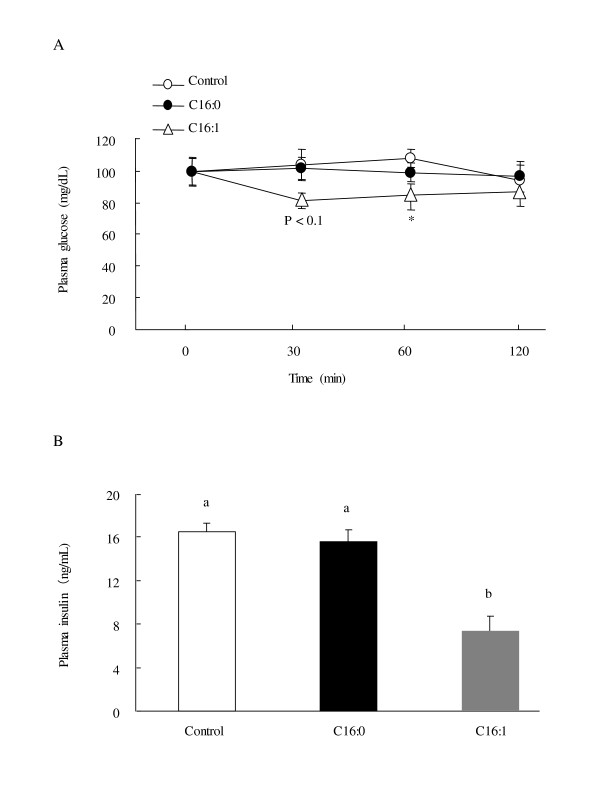
**Effect of palmitoleic acid on insulin resistance in KK-A^y ^mice**. Animals were orally administered vehicle (control), 300 mg/kg of palmitic acid, or 300 mg/kg palmitoleic acid on a daily basis for 4 weeks. (A) Plasma glucose levels in an insulin tolerance test. The insulin tolerance test was carried out on day 22 of the 4-week experiment. Animals were deprived of food for 5 h before administration of the insulin. Blood samples were collected immediately before the insulin injection (0 min) and 30, 60, and 120 min later. Values are means ± SE, n = 10. *p *< 0.1 and *p < 0.05 compared with the control group. (B) Plasma insulin levels. Blood samples were collected at the end of 4-week experiment after over-night fasting. Means in a row with superscripts without common letter are different, *p *< 0.05. C16:0, palmitic acid; C16:1, palmitoleic acid.

When animals were subjected to an insulin tolerance test, plasma glucose levels decreased more rapidly in the palmitoleic acid group than in the control group (Figure 2A). In the palmitoleic acid group, plasma glucose concentrations were lower than in the control group at 60 min (*p *< 0.05) following a single subcutaneous administration of insulin. Plasma glucose levels did not differ between the palmitic acid group and control group. Insulin resistance precedes diabetes onset in KK-A^y ^mice and typically causes higher plasma insulin levels. As shown in Figure 2B, plasma insulin levels were markedly lower (*p *< 0.05) in palmitoleic acid than in the palmitic acid or control group.

### Effect of palmitoleic acid on plasma lipids

Palmitoleic acid treatment significantly decreased plasma levels of triglyceride (*p *< 0.05) (Table 2), when compared to the palmitic acid or control group. No changes were detected, however, in plasma levels of total cholesterol or free fatty acid.

### Effect of palmitoleic acid on liver lipids

**Figure 3:**
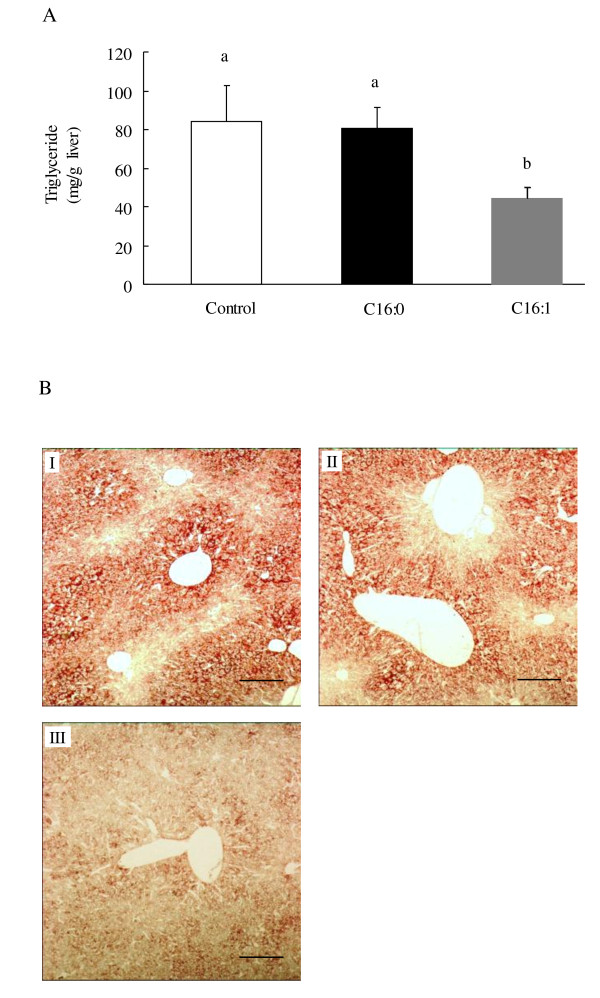
**Effect of palmitoleic acid on hepatic steatosis in KK-A^y ^mice**. Animals were orally administered vehicle (control), 300 mg/kg of palmitic acid, or 300 mg/kg palmitoleic acid on a daily basis for 4 weeks. Graphs depicting neutral lipid accumulation in liver tissue are shown. (A) Hepatic triglyceride concentration. Values are means ± SE, n = 10. Means in a row with superscripts without common letter are different, *p *< 0.05. C16:0, palmitic acid; C16:1, palmitoleic acid. (B) Oil red O staining of liver sections from control (I), palmitic acid (II), and palmitoleic acid (III) groups of KK-A**^y^**mice. Images are shown at 20× magnification. Scale bar = 100 μm.

Administration of palmitoleic acid led to a noticeable decrease (*p *< 0.05) in hepatic triglyceride levels (Figure 3A) compared to either the palmitic acid or control group. Oil red O staining of liver sections revealed neutral lipid content. This analysis indicated that palmitoleic acid reduced hepatic neutral lipid accumulation in KK-A^y ^mice (Figure 3B III) compared to the control group (Figure 3B I). In contrast, no differences were observed between the control and palmitic acid groups (Figure 3B II).

### Effect of palmitoleic acid on mRNA Levels of lipogenic genes in liver

**Figure 4:**
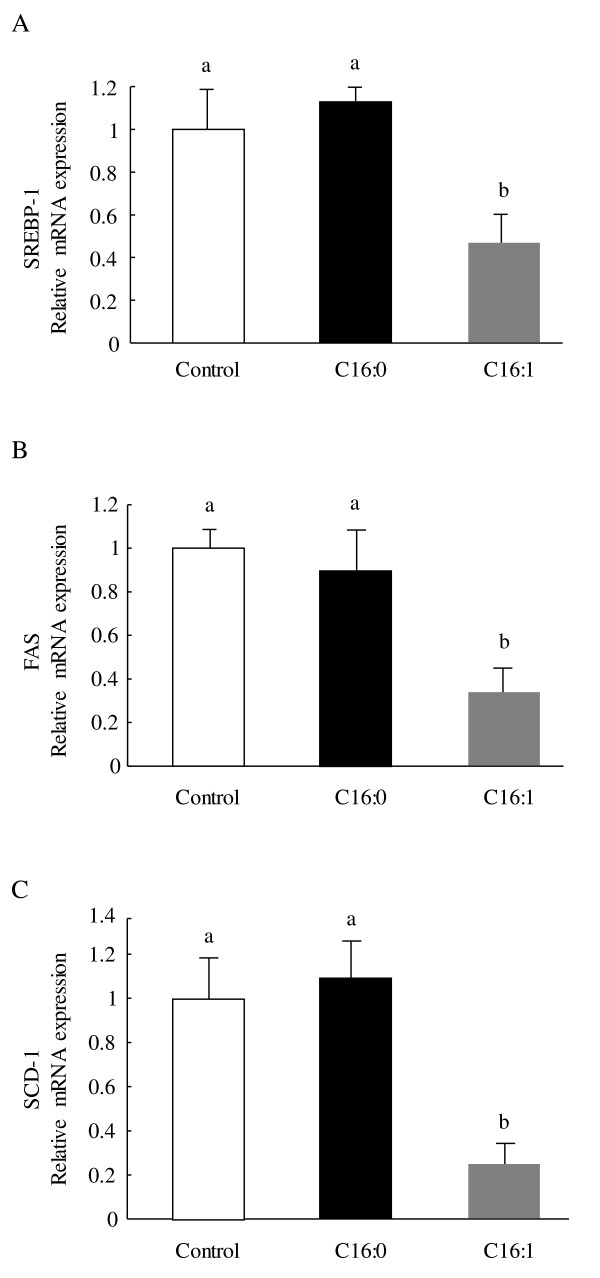
**Effect of palmitoleic acid on lipogenic gene expression in liver in KK-A^y ^mice**. Animals were orally administered vehicle (control), 300 mg/kg of palmitic acid, or 300 mg/kg palmitoleic acid on a daily basis. (A) Relative mRNA expression levels of *SREBP-1*; (B) Relative mRNA expression levels of *FAS*; (C) Relative mRNA expression levels of *SCD-1*. Each value is the mean ± SE, n = 10. Means in a row with superscripts without common letter are different, *p *< 0.05. C16:0, palmitic acid; C16:1, palmitoleic acid; SREBP-1, sterol regulatory element binding protein 1; SCD-1, stearoyl-coenzyme A desaturase 1; FAS, fatty acid synthase.

As shown in Figure 4, *SREBP-1 *(*p *< 0.05), *FAS *(*p *< 0.05), and *SCD-1 *(*p *< 0.05) mRNA levels in the palmitoleic acid group were significantly lower than the respective levels in the palmitic acid group or control group. However, there were no changes in these lipogenic gene mRNA expressions between the palmitic acid and control group.

### Effect of palmitoleic acid on adipocytokine mRNA Levels in adipose tissue

**Figure 5:**
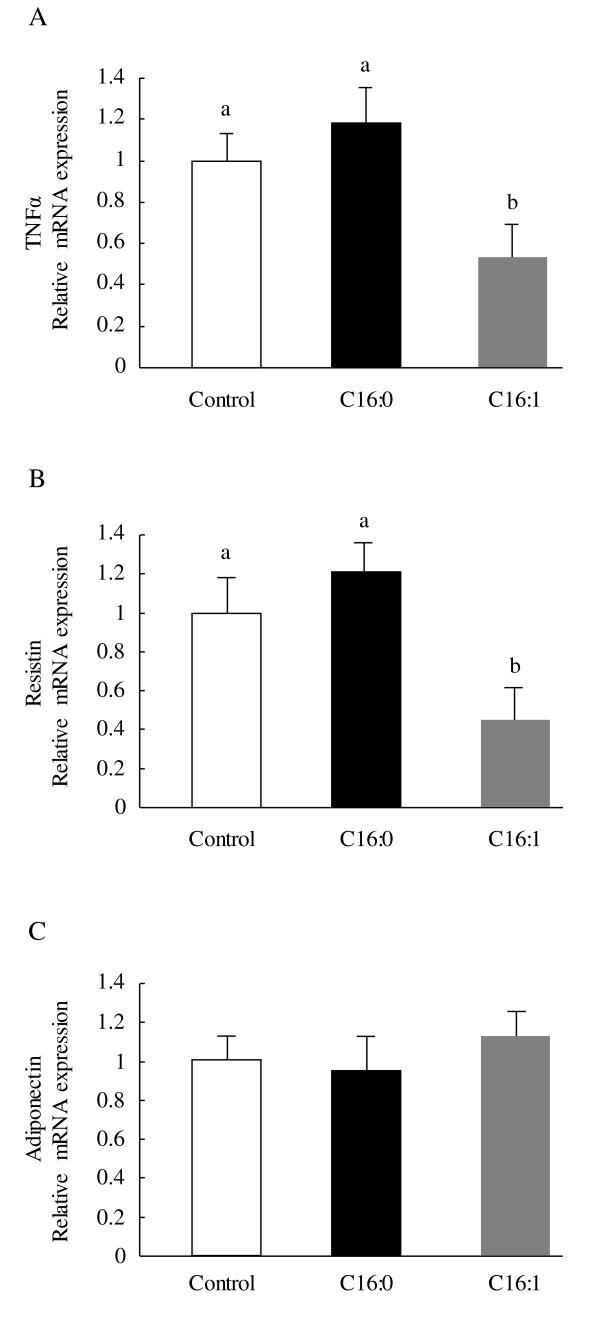
**Effect of palmitoleic acid on adipocytokine mRNA levels in adipose tissue in KK-A^y ^mice**. Animals were orally administered vehicle (control), 300 mg/kg of palmitic acid, or 300 mg/kg palmitoleic acid on a daily basis. (A) Relative mRNA expression levels of *TNFα*; (B) Relative mRNA expression levels of *resistin*; (C) Relative mRNA expression levels of *adiponectin*. Each value is the mean ± SE, n = 10. Means in a row with superscripts without common letter are different, *p *< 0.05. C16:0, palmitic acid; C16:1, palmitoleic acid; TNFα, Tumor necrosis factor alpha.

We measured mRNA levels of adipocytokines that are reported to influence insulin sensitivity (Figure 5). As compared to the palmitic acid or control group, mRNA levels of *TNFα *and *resistin *in mesenteric adipose tissue were markedly suppressed (*p *< 0.05) by the administration of palmitoleic acid for 4 weeks, though no changes in these data were found between the palmitic acid and control group. On the other hand, *adiponectin *mRNA expression levels did not differ between the control and palmitic acid and palmitoleic acid groups.

## Discussion

Our results demonstrate that palmitoleic acid positively affects hyperglycemia, hypertriglyceridemia, and insulin resistance in spontaneously diabetic KK-A^y ^mice. Risk factors for type 2 diabetes mellitus include insulin resistance, hyperglycemia, dyslipidemia, and metabolic syndrome, with insulin resistance being the key underlying metabolic perturbation. Insulin resistance is characterized by reduced responsiveness of target tissues (liver, skeletal muscle, adipocytes) to normal circulating levels of insulin, followed by a progressive decline in insulin secretion from the pancreas [15]. Free fatty acids (saturated fatty acids, in particular) promote insulin resistance and reduce glucose utilization in skeletal muscle [16,17]. However, there are indications that the monounsaturated palmitoleic acid improves glycemic control and increases glucose transport into skeletal muscle cells. This effect is mediated, at least in part, by upregulation of the activities of the glucose transporters GLUT1 and GLUT4 [18,19]. Hyperinsulinemic-euglycemic clamp studies have shown that palmitoleic acid, in the form of a triglyceride, potentiates the insulin-signaling pathway in mice [12]. In the present study, administration of palmitoleic acid, but not palmitic acid, improved insulin resistance. It is likely, therefore, that the effects of palmitoleic acid on hyperglycemia and hypertriglyceridemia can be attributed to improved insulin sensitivity.

To investigate the possible mechanisms behind the beneficial effect of palmitoleic acid on insulin sensitivity, we extended our study to examine adipocytokine gene expression. Numerous studies have shown the close relationship between insulin resistance-linked type 2 diabetes and adipocytokines that are mainly derived from adipose tissue [20,21]. Adiponectin, a key adipocytokine, has been shown to increase insulin sensitivity at least in part through stimulating β-oxidation in skeleton muscle and decreasing hepatic glucose output [22]. However, adiponectin mRNA expression levels did not change by palmitoleic acid administration in the present study, suggesting a minor effect of palmitoleic acid on adiponectin on a gene expression level. On the other hand, repeated administration of palmitoleic acid down-regulated mRNA expressions of TNFα and resistin, the adipocytokines that have been demonstrated to contribute to insulin resistance [23,24]. It is thereby suggested that the beneficial effect of palmitoleic acid on improvement of insulin resistance may be partially owed to suppressing proinflammatory gene expression. In addition, our results also show that pancreas weight increased by repeated administration of palmitoleic acid. In type 2 diabetes, the pancreas fails to produce enough insulin, and evidence suggests that loss of beta cells contributes to this impairment [25,26]. Preventing beta-cell apoptosis and promoting its proliferation, therefore, may represent an important mechanism for improving the diabetic condition. *In vitro *studies have shown that palmitoleic acid prevented the beta cells from high glucose- and palmitic acid-induced impairment of beta-cell proliferation possibly via induction of Bcl-2 [27]. Nevertheless, how palmitoleic acid improves beta-cell function and in turn ameliorates insulin resistance in diabetic model has not been entirely elucidated.

Our present data show that palmitoleic acid suppresses lipid accumulation in the liver. To investigate its mechanism, we assessed liver mRNA levels of genes involved in lipogenesis. Analysis of gene expression with RT-PCR indicated that palmitoleic acid markedly down-regulated mRNA expressions of lipogenic genes such as *SREBP-1*, *FAS *and *SCD-1*. *SREBP-1 *and its target genes *FAS *as well as *SCD-1 *are involved in adipogenesis, and *SREBP-1 *play a central role in regulating fatty acid metabolism [28]. Suppressing effect of palmitoleic acid on hepatic lipid accumulation is thereby possibly due to inhibiting *de novo *lipogenesis. Cao *et al*. [12] demonstrated that fatty acid binding protein-deficient mice have dramatically elevated levels of circulating palmitoleic acid compared to wild-type mice, and that high levels of palmitoleic acid down-regulate genes involved in *de novo *lipogenesis within the liver. It has been reported that hepatic lipid accumulation closely correlates with obesity, insulin resistance, and type 2 diabetes mellitus [29]. In the liver, insulin suppresses hepatic glucose output by inhibiting gluconeogenesis and stimulating glycogen synthesis [30]. In contrast, hepatic steatosis seems to stimulate gluconeogenesis by activating protein kinase C epsilon type and c-Jun N-terminal kinase 1 [31]. These activated proteins subsequently interfere with tyrosine phosphorylation of insulin receptor substance 1 and 2 and impair the ability of insulin to activate glycogen synthase. We therefore infer that palmitoleic acid improves insulin resistance in diabetic mice, at least in part by decreasing hepatic lipid accumulation.

A growing body of evidence indicates that excess body weight is linked to type 2 diabetes [32,33]. Administration of palmitoleic acid to KK-A^y ^mice reduced body weight gain, which may in turn have improved glucose and lipid metabolism. Interestingly, palmitoleic acid treatment resulted in a lower food intake compared with the control group, and studies have shown favorable effects of low calorie intake on obesity-induced metabolic disorders possibly by improving insulin sensitivity [34,35]. Some unsaturated fatty acids, such as the n-3 polyunsaturated eicosapentaenoic acid and docosahexaenoic acid, as well as some short-chain saturated fatty acids, have been shown to regulate secretion of hunger hormones (e.g., the adipose tissue-derived hormone leptin) and gastrointestinal peptides (e.g., glucagon-like peptide-1, cholecystokinin) [36-39]. On the other hand, levels of hunger hormones correlate with energy intake and glucose metabolism [40-42]. The mechanisms by which palmitoleic acid affects food intake, however, remain unresolved.

## Conclusions

In conclusion, oral administration of palmitoleic acid to KK-A^y ^mice dramatically improved their diabetic condition. Body weight increases, hyperglycemia, and hypertriglyceridemia were all reduced in response to palmitoleic acid. In these mice, down-regulation of proinflammatory gene expressions and decreased hepatic lipid accumulation improved insulin sensitivity, which likely led to many of the beneficial effects documented in this study.

## List of abbreviations used

FAS: Fatty acid synthase; RT-PCR: real-time polymerase chain reaction; SCD-1: Stearoyl CoA desaturase-1; SREBP-1: Sterol regulatory element binding protein 1; TNFα: Tumor necrosis factor alpha.

## Competing interests

The authors declare that they have no competing interests.

## Authors' contributions

ZHY participated in the planning, analysis and manuscript preparation. HM participated in the experiment work. AH participated in the planning and organization of the study. All authors read and approved the final manuscript.
