# Correction to: Chronic administration of palmitoleic acid reduces insulin resistance and hepatic lipid accumulation in KK-Ay Mice with genetic type 2 diabetes

**DOI:** 10.1186/s12944-021-01513-w

**Published:** 2021-08-24

**Authors:** Zhi-Hong Yang, Hiroko Miyahara, Akimasa Hatanaka

**Affiliations:** Central Research Laboratory, Tokyo Innovation Center, Nippon Suisan Kaisha, Ltd., 32-3 Nanakuni 1 Chome Hachioji, Tokyo, 192-0991 Japan

**Correction to: Lipids Health Dis 10, 120 (2011)**

**https://doi.org/10.1186/1476-511X-10-120**

Following the publication of the original article [1], it has been found that Fig. 3B has an error of misplacement. In Fig. 3B, the images of Oil red O staining of liver sections from control (I) and palmitic acid (II) had been misplaced. The mistakes were caused by unintentionally misplacing the correct image during figure preparation. The mistakes have been corrected in the revised file Fig. 3B (I) and (II) below. Authors apologize for this error that was caused by unintentional mistake.

**Fig. 3:**
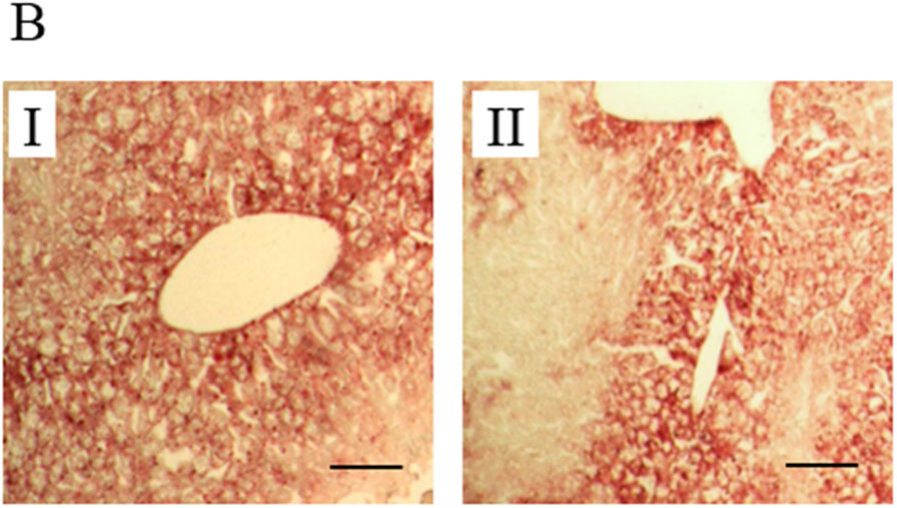
**B**
